# A Holistic Approach to Study Photosynthetic Acclimation Responses of Plants to Fluctuating Light

**DOI:** 10.3389/fpls.2021.668512

**Published:** 2021-04-14

**Authors:** Armida Gjindali, Helena A. Herrmann, Jean-Marc Schwartz, Giles N. Johnson, Pablo I. Calzadilla

**Affiliations:** ^1^Department of Earth and Environmental Sciences, Faculty of Science and Engineering, University of Manchester, Manchester, United Kingdom; ^2^Division of Evolution & Genomic Sciences, Faculty of Biology, Medicine and Health, University of Manchester, Manchester, United Kingdom

**Keywords:** photosynthesis, fluctuating light, mathematical modeling, acclimation, metabolism

## Abstract

Plants in natural environments receive light through sunflecks, the duration and distribution of these being highly variable across the day. Consequently, plants need to adjust their photosynthetic processes to avoid photoinhibition and maximize yield. Changes in the composition of the photosynthetic apparatus in response to sustained changes in the environment are referred to as photosynthetic acclimation, a process that involves changes in protein content and composition. Considering this definition, acclimation differs from regulation, which involves processes that alter the activity of individual proteins over short-time periods, without changing the abundance of those proteins. The interconnection and overlapping of the short- and long-term photosynthetic responses, which can occur simultaneously or/and sequentially over time, make the study of long-term acclimation to fluctuating light in plants challenging. In this review we identify short-term responses of plants to fluctuating light that could act as sensors and signals for acclimation responses, with the aim of understanding how plants integrate environmental fluctuations over time and tailor their responses accordingly. Mathematical modeling has the potential to integrate physiological processes over different timescales and to help disentangle short-term regulatory responses from long-term acclimation responses. We review existing mathematical modeling techniques for studying photosynthetic responses to fluctuating light and propose new methods for addressing the topic from a holistic point of view.

## Introduction

Plants in natural environments are exposed to light and other environmental conditions that fluctuate on timescales ranging over orders of magnitude. The rate of photosynthesis under any given set of conditions will be a function of the light absorbed, the capacity for charge separation in each photosystem, and of the use of that energy to drive carbon assimilation and other metabolic processes. To maximize light capture efficiency at all times, plants need to ensure that the capacities of electron transport and metabolism exceed the maximum rate of light absorption across the full range of environmental conditions experienced. This, however, is unlikely to be the optimal solution overall, in terms of resource allocation between different processes.

Plants growing under different conditions may be limited by, for example, light and water availability, nitrogen and other nutrients, and other abiotic constraints. Plants exposed to low irradiance will tend to invest less in electron transport proteins and enzymes of carbon assimilation, and more in light capture (antenna proteins), aiming to achieve the best photosynthetic performance given the environmental conditions (Anderson et al., 1988; Stewart et al., 2015). Conversely, a drop in temperature will slow down enzymatic activities and diffusion limited processes but will not affect energy absorption or electron transfer. Thus, plants exposed to prolonged low temperature tend to invest more in enzymes, in order to restore the balance between light capture and carbon assimilation (Stitt and Hurry, 2002).

A response to a sustained change in growth conditions over multiple days which involves a change in gene expression is defined as acclimation. Two different types of acclimation can be distinguished: developmental and dynamic (Walters, 2005; Athanasiou et al., 2010). In both, plants adjust their physiology to suit the prevailing environmental conditions. Developmental acclimation includes morphological changes, occurring when tissues develop under different environmental conditions. Dynamic acclimation occurs in fully developed organs, with fixed morphology, and involves changes in protein content and composition, which in turn affects different metabolic fluxes and metabolite concentration. Such alterations ensure optimum resource use under the new condition, and give plants the necessary plasticity to withstand changes in their environment (such as seasonal temperature and moisture changes, light fluctuation, etc.). Following this definition, we can distinguish photosynthetic acclimation from regulation (Herrmann et al., 2019b), the latter encompassing processes that alter the *activity* of particular steps in photosynthesis over a time scale of seconds or/and minutes, without changing the abundance of the proteins involved. It is important to note that regulatory processes may be involved in pathways controlling acclimation and will in turn be affected by the acclimation response itself.

In natural environments and in crop fields, plants receive light energy in the canopy through sunflecks (Pearcy, 1990). The duration and distribution of these are highly variable, impacting the overall photosynthetic yield (Rascher and Nedbal, 2006; Foo et al., 2020). Due to the high frequency of high-low light cycles, responses that avoid photoinhibition and maximize photosynthetic yield are required. Short-term responses to fluctuating light involves almost immediate changes in the thylakoid membranes [e.g., induction of Non-Photochemical Quenching (NPQ), including high energy-state quenching (qE) and state transitions], alteration in the activation state of enzymes (e.g., Benson-Calvin Cycle) and changes in stomatal conductance (Tikkanen et al., 2006, 2010). Meanwhile, long-term acclimation responses might include, amongst others, an increase in the pool size of the xanthophyll cycle pigments and in the PSBS protein content (Wei et al., 2020), which in turn enhance its photoprotective capacity. The interconnection and overlapping of these processes, which can occur simultaneously or sequentially over time, challenge the study of the sensing and signaling pathways involved in long-term fluctuating light acclimation in plants. Thus, an holistic approach is required, to which mathematical modeling techniques can make important contributions.

Systems modeling applies various mathematical techniques to describe and conceptualize the structural and dynamic components of a system, such as a set of biochemical pathways. Mathematical modeling can be applied at different levels and over different time-scales, describing processes inside an organelle, across the whole cell or even multiple tissues (Dada and Mendes, 2011; Gomes de Oliveira Dal'Molin et al., 2015; Shaw and Cheung, 2018). This approach has been extensively applied in biology, including studies of photosynthetic acclimation and regulation of sugar metabolism in plants (Nägele and Weckwerth, 2014; Zakhartsev et al., 2016; Herrmann et al., 2019a, 2020). Mathematical modeling is restricted by the available biological knowledge, and by the assumptions under which that knowledge is synthesized in the model. However, if the model assumptions represent an accurate description of the biological system under study, *in silico* studies can provide insights into the underlying processes that yield experimentally useful information. Often, modeling techniques are employed to generate new hypotheses about a complex system in an efficient, targeted, and cost-effective manner (Kitano, 2002). Thus, mathematical modeling has the potential to disentangle the many observed biochemical changes in a plant's responses to fluctuating environmental conditions to help identify sensors, signals, or acclimation responses.

For the purpose of this review we will consider immediate changes that occur upon changes in light as the inputs of a plant system (i.e., the sensors), and the long-term responses that result from sustained changes in light regimes as the corresponding outputs (i.e., the long-term acclimation responses). Considering the different timescales in which light can effectively fluctuate in natural environments, we will discuss potential signal transduction pathways that could act as links between the inputs and outputs of the system, triggering acclimation. Overall, we aim to gain a deeper understanding on the following questions: How do plants integrate environmental fluctuations over time and how do they tailor their responses accordingly?

## System Inputs: Short-Term Responses to Fluctuating Light

**Figure 1:**
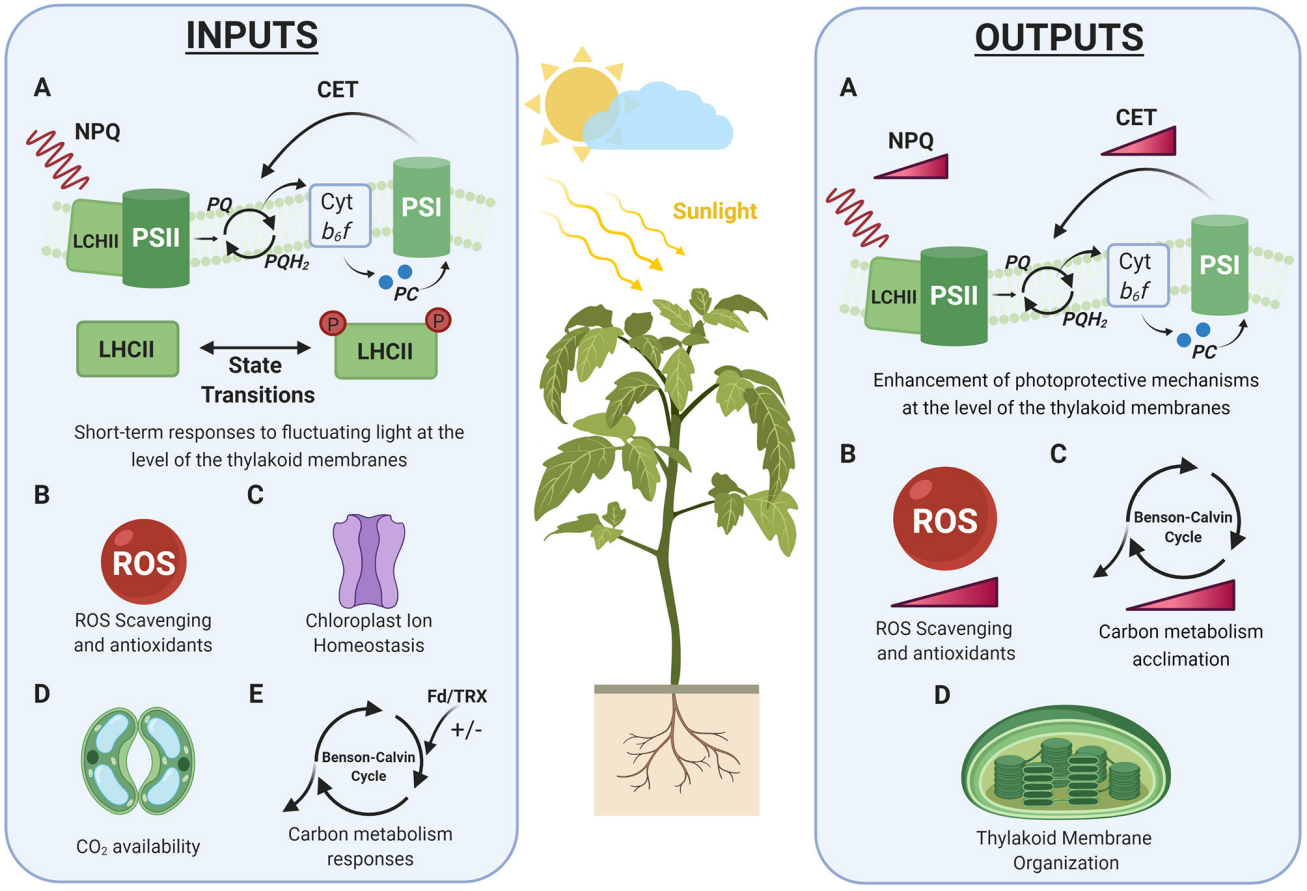
Short and long-term responses to fluctuating light. Schematic representation of the physiological processes defined as inputs and outputs of photosynthetic acclimation to fluctuating light in plants (see sections System Inputs: Short-Term Responses to Fluctuating Light and System Outputs: Long-Term Acclimation to Fluctuating Light). Colored gradient triangles depict accumulation of ROS or intensification of a process. LHCII, Light Harvesting Complex II; PSII, Photosystem II; PSI, Photosystem I; NPQ, Non-Photochemical Quenching; CET, Cyclic Electron Transport; Cyt *b*_6_*f* , Cytochrome *b*_6_*f* ; PQ, Plastoquinone; PQH2, Plastoquinol. Created with BioRender.com.

Fluctuations in irradiance are immediately reflected in the chloroplast and in the physiology of leaves, triggering different short-term responses aimed at maximizing photosynthesis, while protecting the photosynthetic apparatus from photo-oxidative damage (Standfuss et al., 2005; Yamori, 2016). These short-term responses can also be important in triggering long-term acclimation, which is determined not only by the intensity of the incident light, but also by the frequency of oscillations (Qiao et al., 2020). In this section, we will address the different regulatory processes that act as potential inputs for the long-term acclimation responses to fluctuating light in plants (Figure 1).

### Photosynthetic Production of Reactive Oxygen Species (ROS) as a Short-Term Input to the System

Over-reduction of the electron transport chain, which occurs when light absorption exceeds the immediate capacity for CO_2_ fixation, can result in electrons “spilling over” to oxygen, leading to the production of Reactive Oxygen Species (ROS). Most directly, this includes singlet excited oxygen (^1^O_2_), superoxide (${\text{O}}_{2}^{-}$), hydrogen peroxide (H_2_O_2_), and hydroxyl radicals (HO). ROS generation can be triggered in the chloroplast by many environmental factors, including high light, salinity, drought, pathogens, etc. Therefore, plants have evolved a plethora of ROS scavenging mechanisms to minimize the harmful effects of increased ROS levels (Pospíšil, 2012; Foyer, 2018). Although ROS are also generated in other cell compartments, such as mitochondria and peroxisomes, the main sites of ROS production in the chloroplast are photosystem I (PSI) and photosystem II (PSII; Tripathy and Oelmüller, 2012; Pospíšil, 2016).

Oxygen can be reduced to ${\text{O}}_{2}^{-}$ by electrons derived from both photosystems (Zulfugarov et al., 2014; Pospíšil, 2016; Takagi et al., 2016). ${\text{O}}_{2}^{-}$ can be converted to H_2_O_2_ and O_2_, a process that is catalyzed by the enzyme superoxide dismutase (SOD) in the chloroplast stroma (Pospíšil, 2012). H_2_O_2_ can subsequently be converted to water, in the so called water-water cycle, via a series of redox reactions (Awad et al., 2015)_._ Although the scavenging of H_2_O_2_ can act as an alternative electron sink, the electron flux through this pathway is quickly saturated (Driever and Baker, 2011). Thus, excess H_2_O_2_ from the chloroplast can pass to the nucleus, where it modulates gene expression and triggers plant acclimation responses (Figure 1) (Exposito-Rodriguez et al., 2017).

^1^O_2_ is mainly produced via energy transfer from triplet excited chlorophylls (^3^Chl^*^) to oxygen in PSII (Krieger-Liszkay et al., 2008; Pospíšil, 2016). Unlike H_2_O_2_, ^1^O_2_ is believed not to diffuse to the nucleus, due to its short lifetime (~200 ns; Skovsen et al., 2005). However, ^1^O_2_ produced in PSII has been shown to directly react with carotenoids and thylakoid lipids, causing oxidative damage. Oxidative products of carotenoids have been shown to have a signaling role in stress responses (Hideg et al., 1998; Triantaphylidès and Havaux, 2009; Ramel et al., 2012).

### Short-Term Photoprotective Responses

Arguably, the most important photo-protective processes in plants are collectively measured by the parameter Non-photochemical quenching (NPQ). This term includes different components that exhibit distinct activation and deactivation kinetics (Standfuss et al., 2005; Johnson and Ruban, 2009; Niyogi, 2009; Ruban et al., 2012; Derks et al., 2015). The major, and fastest, component of NPQ is high energy state quenching (qE). qE is activated by the formation of a pH gradient (ΔpH) between the thylakoid lumen and the chloroplast stroma, and involves protonation of PSII subunit S (PsbS) and the de-epoxidation of zeaxanthin from violaxanthin through the xanthophyll cycle (Pascal et al., 2005; Takizawa et al., 2007; Ruban et al., 2012).

An increase in light intensity results in more protons being transferred from the chloroplast stroma to the thylakoid lumen via photosynthetic electron flow and, as a result, the pH in the thylakoid lumen drops. Protonation of PsbS leads to conformational changes in LHCII, which in turn increase the amount of energy that is quenched as heat, preventing ROS overproduction and protecting PSII from photodamage (Henmi et al., 2004; Vass, 2012; Zavafer et al., 2015). Under fluctuating light conditions, changes in ΔpH are a transient signal for qE, forming and decaying within seconds. However, the interconversion of violaxanthin and zeaxanthin in response to changes in irradiance occurs over a longer timescale (minutes).

Cyclic electron transport (CET) is another regulatory process involved in photoprotection in plants (Finazzi and Johnson, 2016; Yamori et al., 2016; Yamamoto and Shikanai, 2019), which plays an important role in generating the ΔpH required to trigger qE (Suorsa et al., 2016; Nakano et al., 2019). In CET, electrons are transferred from ferredoxin back to the plastoquinone (PQ) pool, and subsequently to PSI through Cyt *b*_6_*f* and plastocyanin (PC). Thus, CET imports protons from the stroma to the lumen for ATP generation, but without net production of NADPH (Breyton et al., 2006; Joliot and Johnson, 2011). CET helps to balance the ATP:NADPH ratio under circumstances where the rate of consumption of reducing equivalents is reduced. In *Arabidopsis, two* main pathways for cyclic electron flow have been identified (Munekage et al., 2008; Shikanai, 2014). In the antimycin A-sensitive pathway, electrons are transferred from ferredoxin to PQ via a pathway involving the PGR5/PGRL1 complex (Munekage et al., 2002; DalCorso et al., 2008). In contrast, in the antimycin A-insensitive pathway, the electron transfer to the PQ pool is facilitated by NADH:plastoquinone oxidoreductase (NDH; Joliot and Johnson, 2011; Shikanai, 2014). For an extensive review of CET, see Nawrocki et al. (2019).

Of the two pathways of CET, the antimycin A-sensitive pathway has been particularly linked to PSI photoprotection under fluctuating conditions (Suorsa et al., 2012; Yamamoto and Shikanai, 2019). Studies in the *pgr5* mutant of Arabidopsis show that PGR5 participates in photosynthetic control of Cyt *b*_6_*f* (Nandha et al., 2007), protecting PSI from photodamage under low/high light cycles (Suorsa et al., 2012, 2013; Yamamoto and Shikanai, 2019). In addition, PGR5 plays a role in the acceptor-side regulation of PSI. Accordingly, it was recently observed that over-expression of PGR5 in the C4 plant *Flaveria bidentis*, enhances the electron sink downstream of PSI, increasing photoprotection (Tazoe et al., 2020). The water-water cycle has also been suggested as a PSI photoprotective mechanism under fluctuating light conditions, although its activity is strongly species dependent (Huang et al., 2019b; Yang et al., 2020). In this cycle, stromal antioxidants enzymes catalyze ROS conversion into water (Asada, 1999), a process which was recently suggested to be more relevant for PSI photoprotection than CET in angiosperms (Sun et al., 2020).

In addition to qE, another important NPQ component in plants is qT, a form of quenching associated with state transitions (for a review see Minagawa, 2011). This process regulates the distribution of excitation energy between both photosystems, under conditions where the incident light favors the excitation of one over the other. To mitigate such changes, plants can adjust the energy excitation between PSII and PSI within minutes, by altering the distribution of light harvesting proteins between them. The imbalance in the excitation level of the photosystems is sensed through changes in the redox state of the PQ pool (Lemeille and Rochaix, 2010). In particular, reduction of the PQ pool and binding of PQH_2_ at the Qo site of the Cyt *b*_6_*f* , activates a specific kinase (STN7) that phosphorylates LHCII trimers (Wollman and Lemaire, 1988; Vener et al., 1995; Zito et al., 1999; Depège et al., 2003; Bellafiore et al., 2005). Phosphorylation induces LHCII detachment from PSII and partial (or total) attachment to PSI, triggering transition to State II (Kyle et al., 1983; Larsson et al., 1983). Meanwhile, when the PQ pool is oxidized, dephosphorylation of LHCII triggers the opposite phenomenon and transition to State I (Pribil et al., 2010; Shapiguzov et al., 2010). The signals produced from the redox state of the PQ pool are naturally transient, however, evidence shows a direct and rapid regulation of chloroplast gene expression in response to changes in PQ redox state (Pfannschmidt et al., 1999).

Although the classical view of state transitions has been associated with spectral changes in the quality of the incident light, thylakoid phosphorylation can also be triggered dynamically by changes in light intensity (Tikkanen et al., 2010; Grieco et al., 2012; Mekala et al., 2015). While under low white light intensity LHCII phosphorylation levels are maximal, under high light conditions LHCII phosphorylation is down-regulated and PSII core phosphorylation increases (Tikkanen et al., 2010). These opposite states do not change the relative excitation of PSII and PSI, but their regulatory function is related with the maintenance of an equal excitation pressure between both photosystems (Tikkanen et al., 2010). The main kinases and phosphatases involved in this phosphorylation pathway are STN7/STN8 and TAP38/PPH1, respectively; being their regulation particularly relevant under low light conditions (Tikkanen et al., 2010; Mekala et al., 2015). When light intensity increases, other regulatory mechanisms (such as NPQ) become more important for photoprotection (Tikkanen et al., 2010; Grieco et al., 2012). Under fluctuating light conditions, a role for STN7-dependent phosphorylation was also found in PSI photoprotection, through the maintenance of the redox stability of the electron transport chain (Grieco et al., 2012).

Both STN7 and STN8 are also capable of phosphorylating a range of proteins in the chloroplast (Schönberg et al., 2017), extending their involvement in the short-term response to fluctuating light onto further processes of acclimation. The regulatory pathways related with thylakoid protein phosphorylation have a key role in the photosynthetic responses to a changing environment, and their participation in the signals transduction pathway for acclimation needs further elucidation (reviewed by Grieco et al., 2016).

### Changes in Chloroplast Ion Homeostasis

Ion homeostasis in the chloroplast is relevant to light sensing, not only due to its effect on enzymatic activity, but also due to its contribution to the regulation of the proton and electric potentials across the thylakoid membrane (Finazzi et al., 2015). Proton motive force (PMF), the driver of ATP synthesis, consists of two components: ΔΨ, the electrical potential gradient that is built due to ions moving in and out of the thylakoid lumen, and ΔpH. When proton concentration significantly increases in the thylakoid lumen, qE is activated, leading to the loss of energy as heat (Henmi et al., 2004; Vass, 2012; Zavafer et al., 2015). To maintain ATP production without promoting acidification of the lumen, which leads to NPQ activation, fluxes of counter ions (Cl^−^ influx, Mg^2+^ and K^+^ efflux) regulate the ΔΨ component of the PMF (Carraretto et al., 2013; Armbruster et al., 2014; Herdean et al., 2016).

Finetuning ΔpH and ΔΨ to better suit different environmental conditions can facilitate fast modulation of photosynthetic activity under fluctuating light conditions. For instance, a transporter that has been linked to fast photosynthetic regulation in *Arabidopsis* is AtVCCN1, which transports Cl^−^ ions into the chloroplast lumen (Herdean et al., 2016). Influx of Cl^−^ ions into the lumen triggers an increase in the ΔpH/ΔΨ ratio, by decreasing H^+^ efflux from the thylakoid membranes, inducing a faster NPQ response under sudden increases in light intensities. By contrast, potassium influx to the lumen via the K^+^ antiport (KEA3), has been identified as an important factor in the transition from high to low light (Armbruster et al., 2014, 2016; Galvis et al., 2020). KEA3 transfers K^+^ into the lumen and H^+^ out to the chloroplast stroma, decreasing ΔpH but maintaining the ΔΨ necessary for ATP production. KEA3 activity accelerates NPQ relaxation during the transition to low light, leading to a fast recovery of CO_2_ assimilation (Armbruster et al., 2014).

Another K^+^ transporter, the two-pore K^+^ channel (TPK3), has been suggested to play a pivotal role in thylakoid ultrastructure organization and plant growth in *Arabidopsis* (Carraretto et al., 2013). TPK3 exports K^+^ and Ca^+^ ions, and is thought to modulate fast regulation of PMF to optimize photosynthetic activity under different light environments (Carraretto et al., 2013). However, recent results obtained by Höhner et al. (2019) showed that TPK3 is localized in the tonoplast and is not involved in photosynthetic regulation. These authors suggest the involvement of an as yet unknown additional K^+^ channel in photosynthetic acclimation to fluctuating light.

### Thioredoxins as Signals for Light

The light-induced enzymatic activation of the Benson-Calvin cycle was first discovered by Buchanan and colleagues in the 1960s, showing that CO_2_ fixation was activated by light (reviewed by Buchanan et al., 2002; Michelet et al., 2013). This light activation pathway, called the ferredoxin/thioredoxin (Fd/TRX) system, regulates carbon metabolic pathways through post-translational redox modifications (reviewed by Ruelland and Miginiac-Maslow, 1999; Lemaire et al., 2007; Michelet et al., 2013; Nikkanen et al., 2017). Thioredoxins (TRX) in the chloroplast are reduced mainly by ferredoxin (Fd, the PSI electron acceptor), via an enzyme called Ferredoxin-Thioredoxin reductase (FTR). Once reduced, TRX can reduce disulfide bonds in different stromal target proteins, placing the Fd/TRX at the crossroads between the “light” and “dark” reactions of photosynthesis (Ruelland and Miginiac-Maslow, 1999; Lemaire et al., 2007).

Activation of the Fd/TRX system will directly depend on the redox state of the chloroplast, meaning that changes in the photosynthetic electron flow will activate/deactivate different target enzymes under changing light regimes. This on/off switch acts as a significant regulatory process, leading to the adjustment of the carbon metabolism under different conditions. However, most studies on the regulatory role of Fd/TRX have been conducted under continuous light conditions, and research on their involvement in fluctuating light responses is limited (Collin et al., 2004; Nikkanen and Rintamäki, 2014; Geigenberger et al., 2017).

A study performed on the Arabidopsis knockout mutants t*rxm1/m2* showed the role of thioredoxins in the short-term responses to fluctuating light (Thormählen et al., 2017). Mutant plants showed alterations in the light activation of the enzyme malate dehydrogenase (MDH) and the malate/oxaloacetate (Mal/OAA) shuttle, a higher NPQ and a lower PSII quantum efficiency. This phenotype was only evident under fluctuating light conditions, with these alterations being more pronounced with increasing numbers of high-low light cycles. By contrast, no phenotypic differences were seen between the mutants and the WT plants under constant light.

### Impact of Fluctuating Light on CO_2_ Availability and Photorespiration

Stomatal responses play a critical role in the availability of CO_2_ for carbon fixation, and it has been shown that stomatal dynamics limit photosynthesis under fluctuating light (Qu et al., 2016; Papanatsiou et al., 2019; De Souza et al., 2020; Kimura et al., 2020). Since stomatal responses are slower than photochemical and biochemical regulatory changes, a sudden change in light intensity could cause chloroplast CO_2_ concentration to decrease (Huang et al., 2015; Vialet-Chabrand et al., 2017). A decrease in CO_2_ concentration (and thus carbon fixation) implies that fewer electrons are being directed to the Benson-Calvin cycle, favoring the over-reduction of the electron transport chain and triggering ROS generation. At the same time, a decrease in CO_2_ availability also increases O_2_ binding to Rubisco, its oxygenase activity and photorespiration (Huang et al., 2015). Interestingly, it was recently shown that stomatal opening and closure dynamics can acclimate to different growth light regimes, anticipating future variations in light, and adjusting CO_2_ availability to the prevailing light condition (Matthews et al., 2018).

Photorespiration is a metabolic pathway that recycles 2-phosphoglycolate (2PG), a toxic product of the oxygenase activity of Rubisco, into 3-phospholycerate (3PGA; reviewed by Foyer et al., 2009; Bauwe et al., 2010; Eisenhut et al., 2019). This recycling requires several enzymatic steps that are distributed across three different organelles: the chloroplast, the peroxisome, and the mitochondrion. Although the photorespiratory pathway can represent a substantial loss of CO_2_ fixation, its involvement in photoprotection, nitrogen assimilation, and abiotic stress responses make it a crucial process for plants (reviewed by Foyer et al., 2009; Bauwe et al., 2010; Timm and Bauwe, 2013; Voss et al., 2013; Eisenhut et al., 2019). Nevertheless, its participation under fluctuating light conditions has not been extensively studied (Huang et al., 2015; Schneider et al., 2019). Huang et al. (2015) showed that, under fluctuating light conditions, a strong activation of the photorespiratory pathway allows the consumption of reducing equivalents, decreasing the reduction pressure of the electron transport chain and avoiding ROS generation. In addition, RuBP regeneration is also accelerated, favoring carbon fixation under these circumstances.

## System Outputs: Long-Term Acclimation to Fluctuating Light

Acclimation to environmental fluctuations involves changes in gene expression and protein abundance, which result in the modification of the structure and composition of tissues. In particular, dynamic acclimation occurs in developed tissues, constrained by the existing structures, and involves processes or responses that take several days to be achieved. These responses might depend on the plant species and on the intensity and duration of the environmental fluctuation (Yin and Johnson, 2000). The processes involved in dynamic acclimation are not necessarily irreversible, and they will persist as long as the prevailing environmental condition is maintained. In the following sections, we will focus on the changes involved in the dynamic acclimation of photosynthesis under fluctuating light conditions (Figure 1).

### Acclimation of Carbon Metabolism

When plants are grown at higher irradiances, they typically develop leaves with a high capacity for photosynthesis (see Walters, 2005). Fully developed leaves transferred from low to high light can also increase their photosynthetic capacity, typically over a period of a week (Athanasiou et al., 2010; Dyson et al., 2015). This acclimation response involves extensive changes across the whole of the leaf proteome, with marked increases in the concentration of Rubisco and other enzymes involved in the Benson-Calvin cycle, as well as down-stream enzymes involved in carbon assimilation (Miller et al., 2017).

Schneider et al. (2019) observed an upregulation of some Benson-Calvin cycle enzyme genes, such as fructose-1,6-bisphosphate aldolase 1 (FBA1) and sedoheptulose-1,7-bisphosphatase (SBSPASE), in Arabidopsis plants subjected to fluctuating light for 3 days. These enzymes were previously shown to participate in the regulation of the metabolic flux of carbon in plants (Lefebvre et al., 2005; Uematsu et al., 2012; Simkin et al., 2015, 2017). In addition, SBSPASE and FBA1 were also found to be regulated by the Fd/TRX system (Breazeale et al., 1978; Sahrawy et al., 1997; Dunford et al., 1998), suggesting a fine-tuning regulation of this long-term acclimation by a short-term mechanism. However, despite the increased activity and/or concentration of their Benson-Calvin enzymes, when compared to constant light conditions, plants under fluctuating light do not necessarily show an enhancement of their CO_2_ fixation capacity (Watling et al., 1997; Vialet-Chabrand et al., 2017; Schneider et al., 2019). Studies show that proteomic and transcriptomic changes in response to fluctuating light do not always align, suggesting a role of post-transcriptional regulations in the modulation of long-term acclimation responses (Athanasiou et al., 2010; Dyson et al., 2015; Miller et al., 2017; Schneider et al., 2019; Niedermaier et al., 2020).

Furthermore, as part of the acclimation response of carbon metabolism to fluctuating light, an increase in the expression of photorespiratory genes, and their corresponding protein content, was also observed in Arabidopsis (Schneider et al., 2019; Niedermaier et al., 2020). This metabolic response was shown to be particularly significant under high light fluctuation periods (Huang et al., 2015). Under low light fluctuating regimes, an increase in the photorespiratory pathway was deemed insignificant (Kono et al., 2014), possibly due to a lower accumulation of reducing equivalents.

### Thylakoid Membrane Changes During Long-Term Acclimation

In addition to metabolic alterations, changes in the thylakoid membrane protein composition play an important role in light acclimation (reviewed by Walters, 2005; Anderson et al., 2012; Kaiser et al., 2018; Johnson and Wientjes, 2020). For instance, plants grown under high light have been observed to have a lower PSII/PSI ratio, but higher concentrations of Cyt *b*_6_*f* and ATPase (reviewed by Evans, 1988; Eskins et al., 1991; Walters and Horton, 1994; Bailey et al., 2001; Walters, 2005). Furthermore, high light may also reduce the amount of LHCII and increase the chlorophyll *a/b* ratio, which is related with changes in light harvesting complexes concentration and photosystems ratio (Leong and Anderson, 1984; Yang et al., 1998; Bailey et al., 2001). By contrast, LHCII concentration increases when light exposure is limiting for plant growth, although under these conditions, a compensating decrease in PSII levels is also observed (Evans, 1988; Bailey et al., 2001). Some, but not necessarily all, of these responses are seen when plants are exposed to step changes in irradiance. In Arabidopsis, transfer from low to moderately high light resulted in an increase in Cyt b_6_f and ATPase, without measurable changes in chlorophyll content or the total amount of LHC proteins (Athanasiou et al., 2010; Miller et al., 2017).

In addition to acclimation to overall light intensity, when plants are exposed to different light *qualities*, the protein composition of the thylakoid membranes may also change (reviewed by Anderson et al., 1988). Long-term acclimation responses include changes in LHCII concentration, and Chl *a*/*b* and PSII/PSI ratio (Chow et al., 1990; Kim et al., 1993; Walters and Horton, 1995; Murchie and Horton, 1998). Such changes, occurring within days; help balance the electron transport rate under situations where either photosystem is preferentially excited by light. Importantly, these alterations differ from state transitions, which occur in seconds to minutes and do not include changes in thylakoid membrane composition, only re-distribution of LHCII and photosystem macro-organization.

Overall, when light intensity or quality change, plant acclimation responses tend to balance light absorption and assimilation (Rott et al., 2011; Yamori et al., 2011). Nevertheless, understanding the effect of fluctuating light in natural environments is far more complex. Sunflecks will have a direct impact on light intensity, but natural shade cast by vegetation will also affect the incident light quality. Thus, natural light fluctuations in the ecosystems result in complex inputs, which might induce contradictory output responses. For instance, the PSII/PSI ratio will change in opposite directions with a decrease in light intensity or with exposure to a high far-red/red ratio, conditions that can be imposed by vegetative shading (Murchie and Horton, 1998; Bailey et al., 2001). Consequently, predicting a thylakoid membrane specific response to fluctuating light in natural environments is not an easy task. Furthermore, many of these responses are species-dependent (Murchie and Horton, 1998; Yin and Johnson, 2000).

### Enhancement of the Photoprotective Mechanisms as a Long-Term Acclimation Response

Similar to what happens following sudden increases in light intensity, frequent exposure to oscillating periods of high light induces over-reduction of the electron transport chain, triggering an increase in ROS production and photoinhibition (Shimakawa and Miyake, 2018; Huang et al., 2019a). Thus, long-term acclimation to fluctuating light can also involve an enhancement of photoprotective mechanisms. For instance, Schneider et al. (2019) observed an up-regulation of H_2_O_2_ scavenging enzymes, such as glutathione peroxidase (GPX7) and catalase (CAT2), in Arabidopsis leaves exposed to 3 days of fluctuating light. In agreement, these plants also increased their ascorbate pool size, indicating an improvement in ROS scavenging and antioxidant response (Schneider et al., 2019).

Fluctuating light can increase PSBS content and the concentration of pigments of the xanthophyll cycle, leading to a strengthening of the photoprotective capacity of NPQ (Barker et al., 1997; Niinemets et al., 1998; Alter et al., 2012; Caliandro et al., 2013). This acclimation response was demonstrated to be, at least partially, regulated at the transcriptional level (Schneider et al., 2019). The relevance of NPQ as a long-term acclimation response to fluctuating light was recently shown using tobacco transgenic lines overexpressing PSBS and zeaxanthin epoxidase (ZEP) and violaxanthin de-epoxidase (VDE), the key enzymes in the xanthophyll cycle (Kromdijk et al., 2016). These plants showed a higher CO_2_ assimilation compared to the WT, leading to a higher dry mass accumulation under field conditions.

In addition to NPQ, genes related to CET were also upregulated in response to fluctuating light (Schneider et al., 2019). Proteomic results obtained under the same conditions, and by the same authors, suggest a specific role of the NDH-like complex in this long-term acclimation mechanism (Niedermaier et al., 2020). Nevertheless, mutants lacking PGR5 were shown to suffer strong PSI photoinhibition under fluctuating light (Suorsa et al., 2012; Kono and Terashima, 2016), meaning that the involvement of the antimycin A-sensitive CET pathway in this acclimation process cannot be ruled out. It is worth mentioning that a higher CET flux will contribute to a higher ΔpH, facilitating NPQ generation under photoinhibitory conditions (Munekage et al., 2002). Thus, the involvement of CET in this long-term acclimation response will not only avoid PSI photoinhibition, but also increase thermal dissipation. Consequently, short-term responses to sudden increases in light intensity are also being improved by this long-term response.

## Signals Transduction Pathways for Long-Term Acclimation to Fluctuating Light

**Figure 2:**
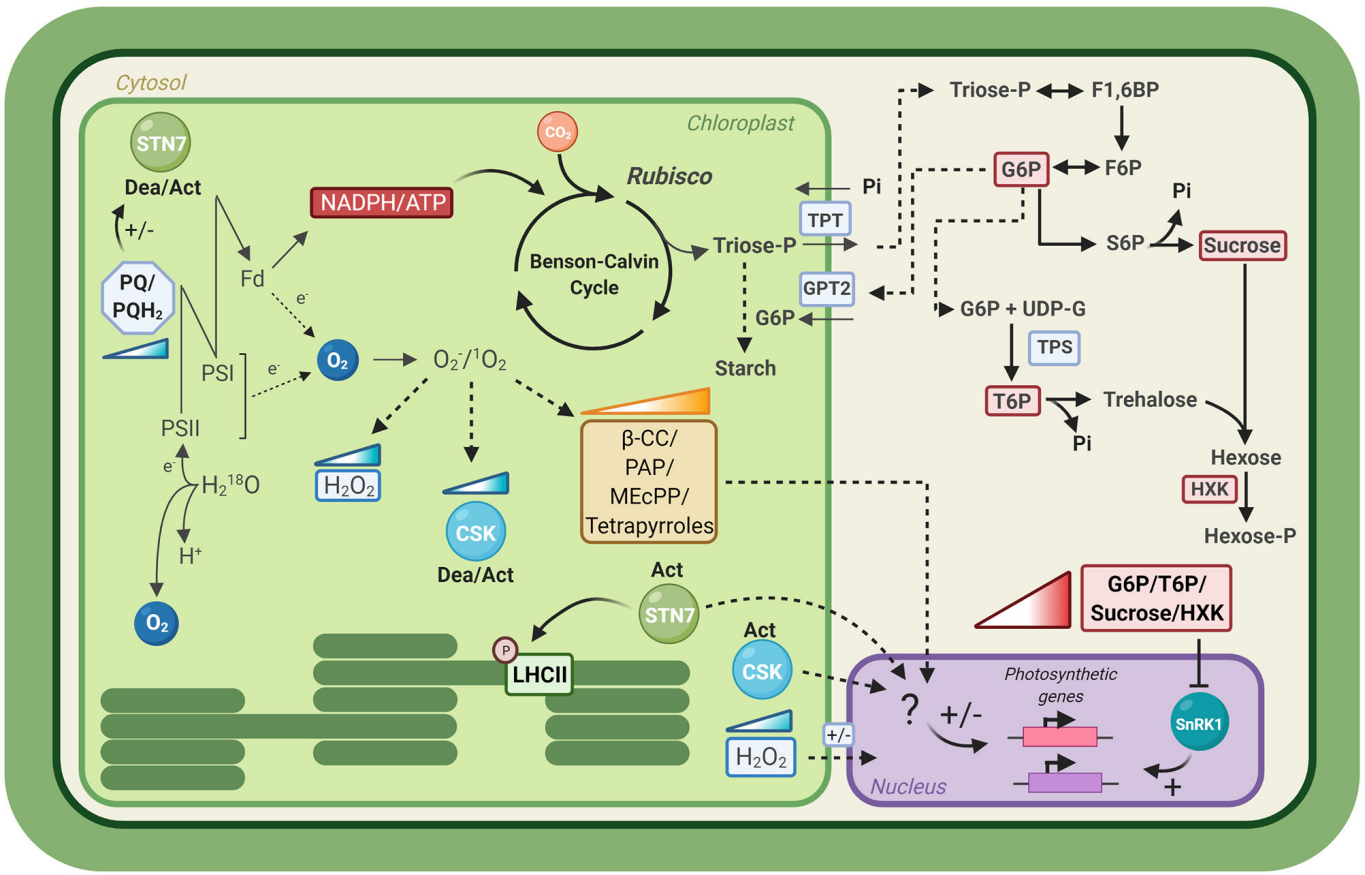
Signals transduction pathways of long-term acclimation to fluctuating light. Schematic representation of the different putative signaling pathways involved in photosynthetic acclimation to fluctuating light, as described in section Signals Transduction Pathways for Long-Term Acclimation to Fluctuating Light. Colored gradient triangles depict accumulation of a metabolite or enzyme. LHCII, Light Harvesting Complex II; PSII, Photosystem II; PSI, Photosystem I; Fd, Ferredoxin; PQ, Plastoquinone; PQH2, Plastoquinol; STN7, STN7 kinase; CSK, Chloroplast Sensor Kinase; SnRK1, SNF1-related kinase 1 β-CC, β-cyclocitral; PAP, 3′-phosphoadenosine 5′-phosphate; MEcPP, methylerythritol cyclodiphosphate; TPT, Triose-phosphate transporter; GTP2, glucose 6-phosphate/phosphate translocator; G6P, glucose-6-phosphate; T6P, trehalose-6-phosphate; F6P, fructose-6-phosphate; S6P, sucrose-6-phosphate; F1,6BP, fructose-1,6-biphosphate; TPS, Trehalose phosphate synthase; HXK, Hexokinase; Dea/Act, Deactivation/Activation. Created with BioRender.com.

Short-term input responses, such as redox changes in the photosynthetic apparatus, occur on very rapid timescales (μsec-min), close to those of the natural fluctuations of the light environment. These inputs may trigger cellular changes, such as protein phosphorylation or thiol reductions, which respond more slowly to the changing conditions (minutes). Nevertheless, all these inputs are transient, and so, their putative role in signaling for long-term plant acclimation, which occurs over days, is not obvious. The concentrations of metabolites can also change on rapid timescales, in direct response to changing light conditions; but in some cases their accumulation provides the potential to generate signals which average out the short-term fluctuations in the environment. In the following section, we will address some of the most discussed pathways participating in photosynthetic acclimation to fluctuating light in plants (Figure 2).

### ROS and the Redox State of the Chloroplast

Retrograde signals originating from photosynthesis have been studied extensively, aiming to describe chloroplast-nucleus communication and regulation of gene expression (Pfannschmidt et al., 1999; Fey et al., 2005b; Wilson et al., 2006; Foyer et al., 2012; Karpiński et al., 2013; Gollan et al., 2015; Matsubara et al., 2016; Leister, 2019). Within these signaling pathways, the participation of ROS originating from photosynthetic electron flow, has been widely discussed (Pfannschmidt et al., 2009; Ramel et al., 2012; Szechyńska-Hebda and Karpiński, 2013; Kim, 2020). For instance, H_2_O_2_ can diffuse from the chloroplast, creating a signal cascade ultimately affecting nuclear gene expression (Maruta et al., 2012). Meanwhile, oxidation of β-carotene by ^1^O_2_ has been shown to lead to the formation of the volatile compound β-cyclocitral (β-CC), which triggers changes in nuclear gene expression under stress conditions (Ramel et al., 2012; Havaux, 2014; Tian, 2015). Nevertheless, the specific factors participating in this ROS-related signal transduction pathways are still far from being completely elucidated (reviewed by Leister, 2019; Kim, 2020).

In addition, we can think the chloroplast as an environmental sensor for plants, with the redox state of the electron transport chain being a key gear for sensing fluctuations in the environment. Amongst the components of the electron transport chain, the involvement of the PQ pool in retrograde signaling has been suggested, facilitating a rapid physiological response to changing light conditions (El Bissati and Kirilovsky, 2001; Fey et al., 2005a; Bräutigam et al., 2009). Different proteins have been shown to be regulated by the redox state of PQ, and in a ROS-independent manner (Adamska and Kloppstech, 1991; Kimura et al., 2003; Yabuta et al., 2004), and a link between the redox state of the PQ pool and photosystem gene expression has even been observed (Pfannschmidt et al., 1999; El Bissati and Kirilovsky, 2001).

Although it is clear that ROS retrograde signaling and chloroplast redox changes participate in light acclimation, it is still not yet understood how these signals are integrated under fluctuating light. The short lifetime of these chloroplast redox changes imply that other factors might be integrating the redox variations over time. Puthiyaveetil et al. (2008) suggested that a key protein participating in retrograde signaling is the Chloroplast Sensor Kinase (CSK) (Figure 2; Puthiyaveetil et al., 2008). This protein is widely seen in photosynthetic organisms and has been shown to bind an iron-sulfur cluster, which can sense changes in the chloroplast redox state (Ibrahim et al., 2020). The activation/deactivation of the kinase regulates the expression of different photosynthetic genes (Puthiyaveetil et al., 2008; Ibrahim et al., 2020), supporting its participation in the adjustment of the stoichiometry of photosynthetic complexes under different light conditions. Interestingly, CSK gene expression was found to be upregulated under fluctuating light in Arabidopsis (Schneider et al., 2019).

### STN7 Kinase as a Mediator Between the Chloroplast-Nucleus Communications

The STN7 kinase phosphorylates LHCII and some PSII subunits upon changes in light conditions, triggering state transitions and regulating PSII turnover (Bellafiore et al., 2005; Tikkanen et al., 2006, 2010; Wagner et al., 2008; Pietrzykowska et al., 2014). Although STN7 involvement in short-term regulatory responses to fluctuating light has been well-described, it was also shown that *stn7* mutants are unable to undergo forms of long-term acclimation under changing light regimes (Bonardi et al., 2005; Pesaresi et al., 2009). When *stn7* mutants were grown under fluctuating light they exhibited reduced growth and a lower seed yield and were incapable of adjusting their thylakoid composition to the conditions experienced. In agreement, STN7 gene expression was increased after 3 days of fluctuating light in Arabidopsis (Schneider et al., 2019), again highlighting its potential involvement in long-term acclimation. Thus, STN7 seems to play a critical role in both short- and long-term responses to fluctuating light regimes in plants, either directly or indirectly (Bonardi et al., 2005; Wagner et al., 2008; Bräutigam et al., 2009; Leister, 2019).

Although it is still unknown which proteins participate downstream of STN7 in the long-term acclimation response, one of the putative proteins is TSP9. TSP9 is a plant specific nuclear-encoded protein, found in the thylakoid membranes, which is phosphorylated by STN7 upon illumination (Carlberg et al., 2003; Zer and Ohad, 2003). The phosphorylated form dissociates from the thylakoid membrane and has been proposed to act as a signaling molecule regulating gene expression under changing light conditions (Carlberg et al., 2003; Zer and Ohad, 2003; Fristedt et al., 2009). Nevertheless, downregulation of TSP9 in Arabidopsis plants did not affect the long-term response to light changes (Pesaresi et al., 2009), but its mutation was shown to affect state transitions and NPQ (Fristedt et al., 2009). These results suggest the involvement of TSP9 in the short-term responses to fluctuating light, but not in long-term acclimation. The downstream factors involved in the STN7 long-term acclimation signaling pathway, are still elusive (Leister, 2019).

### Metabolites Accumulation as Putative Integrative Factors of the Long-Term Acclimation Response to Fluctuating Light

#### ROS-Related Metabolites

A putative role for signal integration was suggested for the volatile compound β-cyclocitral (β-CC; Ramel et al., 2012; Havaux, 2014; Tian, 2015); the accumulation of 3′-phosphoadenosine 5′-phosphate (PAP) and its regulation by the SAL1 phosphatase (Estavillo et al., 2011; Chan et al., 2016); and the isopropanoid precursor methylerythritol cyclodiphosphate (MEcPP; Xiao et al., 2012). The accumulation of chloroplast tetrapyrrole biosynthesis intermediates was also suggested to be involved in retrograde signaling (reviewed by Nott et al., 2006; Tabrizi et al., 2016), although this model has been questioned (Mochizuki et al., 2008; Moulin et al., 2008).

The accumulation of these metabolites is directly connected to an increase in ROS production and/or redox changes in the chloroplast (Figure 2), and its involvement in the acclimation response to different stress conditions has been described (Estavillo et al., 2011; Ramel et al., 2012; Xiao et al., 2012; Chan et al., 2016). Thus, it is feasible to think that a putative increase in their accumulation over a certain threshold might also trigger an acclimation response to fluctuating light, allowing the integration of different signals over time. This hypothesis is an interesting starting point to close the gap between the short and long-term responses to this environmental condition.

#### Carbon-Related Metabolites

##### Sucrose and the Availability of Inorganic Phosphate

The significance of carbon metabolites as signals for acclimation relies on the fact that photosynthate partitioning varies with changes in irradiance and photoperiod (Mengin et al., 2017). Furthermore, different studies support a role for the accumulation of some of these carbon sinks in the plant's response to several environmental conditions. For instance, sucrose shows a significant role in cold acclimation, triggering changes in gene expression and anthocyanin biosynthetic pathways (Solfanelli et al., 2006; Rekarte-Cowie et al., 2008). In addition, evidence supports a sucrose signaling role in many developmental processes in the plant's life cycle (reviewed by Horacio and Martinez-Noel, 2013).

Sucrose concentrations in plant tissues correlate with light intensity, and their synthesizing enzymes fluctuate over the photoperiod (Cheikh and Brenner, 1992; Horacio and Martinez-Noel, 2013). In addition, sucrose downregulates CO_2_ fixation through alteration of gene expression (Pamplin and Chapman, 1975; Sheen, 1990; Rook et al., 1998; Wiese et al., 2004), and through a negative feedback regulation due to inorganic phosphate (Pi) availability (Hurry et al., 2000; Ensminger et al., 2006). Sucrose synthesis and degradation participate in Pi cycling between the chloroplast and the cytosol (Hurry et al., 2000). Alterations in sucrose synthesis in the cytosol may decrease Pi availability in the chloroplast, inhibiting ATP synthesis and, as a consequence, RuBP regeneration and carbon fixation (Hurry et al., 2000; Ensminger et al., 2006).

Due to its constitutive presence in the cytosol of plant cells (Figure 2), a role for sucrose as a signaling molecule in photosynthetic acclimation may imply that its accumulation needs to exceed a certain threshold (Horacio and Martinez-Noel, 2013). Alter et al. (2012) analyzed the concentration of soluble sugars (glucose, fructose, and sucrose) in Arabidopsis plants under constant and fluctuating light conditions, without observing any differences in total soluble sugar concentration between treatments. Nevertheless, the individual concentration of each sugar was not independently assessed, and changes in sugar ratios under fluctuating light cannot be ruled out.

##### Glucose-6-Phosphate, Trehalose-6-Phosphate and Hexokinases, Key Molecules in the Starch and Sucrose Synthesis Regulation

Photosynthesis, through the Benson-Calvin cycle, produces glycerate-3-phosphate, which is reduced to triose-phosphate (triose-P) in successive reactions that consume NADPH and ATP. Triose-P can be used to regenerate ribulose-1,5-*bis*phosphate (RuBP) in the Benson-Calvin cycle, or, when in excess, can be transformed to end products such as sucrose or/and starch (Figure 2; reviewed by Ensminger et al., 2006). Sucrose is synthesized in the cytosol, for which triose-P is exported from the chloroplast through the triose-phosphate translocator (TPT; Figure 2). When the rate of triose-P export is lower than its rate of synthesis, starch is synthesized in the chloroplast (Zeeman et al., 2004). Carbon flux to starch is also an important strategy to avoid carbon sink limitations under photoinhibitory conditions (Ensminger et al., 2006).

Fixed carbon may also be imported back from the cytosol in the form of glucose-6-phosphate (G6P), through the glucose 6-phosphate/phosphate translocator GPT2 (Figure 2; Niewiadomski et al., 2005; Dyson et al., 2015). GTP2 expression is known to be associated with alterations in carbon metabolism and high light responses, leading to photosynthetic acclimation (Athanasiou et al., 2010; Kunz et al., 2010; Dyson et al., 2015). GTP2 might directly affect the relative concentrations of G6P between cell compartments, affecting the metabolic signals triggering photosynthetic responses to different environmental stimulus. For instance, G6P positively regulates sucrose synthesis, by activating SPS and inhibiting sucrose synthase (SUS- an enzyme participating in sucrose catabolism). This inhibition in sucrose degradation is through inhibition of SNF1-related kinase 1 (SnRK1), which participates in the regulation of carbon metabolism, ABA signaling, stress responses and development (Jossier et al., 2009; Zhang et al., 2009; Cho et al., 2012). Thus, regulating the G6P concentration in the cytosol might have a direct impact on metabolism, growth and acclimation under different environmental conditions.

In a similar way to G6P, trehalose-6-phosphate (T6P) has been described as having an important role in the regulation of carbon assimilation and sugar status in plants (reviewed by Ponnu et al., 2011). T6P is an intermediate in trehalose biosynthesis, synthesized from UDP-Glucose and G6P in the cytosol, by the enzyme trehalose phosphate synthase (TPS; Figure 2; Häusler et al., 2014). T6P is a signal of sucrose availability, alters the rate of starch biosynthesis in the chloroplast and participates in the cross-talk of metabolic regulations through inhibition of SnRK1 (Lunn et al., 2006; Zhang et al., 2009; Yadav et al., 2014). In particular, T6P was also shown to down-regulate genes related to the photosynthetic process, which are normally up-regulated by SnRK1 (Zhang et al., 2009).

Other proposed sugar sensing molecules are the hexokinases (HXKs), which catalyze the phosphorylation of glucose and fructose and have been defined as evolutionarily conserved glucose sensors (Figure 2; reviewed by Granot et al., 2014). HXKs are able to down-regulate the expression of photosynthetic genes, reduce chlorophyll levels and photosynthetic rates (Jang et al., 1997; Dai et al., 1999; Xiao et al., 2000). As a consequence, HXKs are capable of modulating photosynthesis in a glucose dependent-manner, integrating short-term changes in the environment with their corresponding photosynthetic responses (Moore et al., 2003). In agreement, within guard cells, HXK also regulates stomatal closure, supporting a negative coordinated regulation of photosynthesis by hexose availability (Kelly et al., 2013; Granot et al., 2014).

## Metabolic Modeling in Unraveling the Photosynthetic Acclimation to Fluctuating Light in Plants

Our review of the literature shows that experimental studies that link rapid responses to sustained long-term changes are rare, as they are laborious and often technically infeasible. Mathematical modeling, however, has the potential to overcome some of these limitations, helping to identify mechanisms by which plants integrate short-term responses to the environment over time. A holistic understanding of fluctuating light acclimation is a challenge, involving many timescales. The following section covers a range of mathematical modeling techniques that have previously been applied to study photosynthesis (Table 1), and further proposes new modeling techniques that could be employed to deepen our understanding of photosynthetic acclimation to fluctuating light in plants.

**Table 1:** A brief overview of the primary types of models applied to study photosynthetic responses to fluctuating light.

**Type of model**	**Description**	**Advantages**	**Limitations**	**Examples**
Empirical	Statistical methods are used to identify consistently reoccurring patterns in data	No prior knowledge of the underlying biological processes is required	Dependent on high amounts of input data	Stegemann et al., 1999; Louarn et al., 2015
Mechanistic	Systems are broken down into smaller components whose interactions with one another are clearly defined	Can be generalized and used to predict outcomes outside of the range of the input data	Knowledge of the workings of the systems components is required	Farquhar et al., 1980; Kirschbaum et al., 1997; Pearcy et al., 1997
Dynamic	Models mainly consisting of ordinary of partial differential equations that capture changes over time	Can incorporate changes in concentrations over time as well as kinetic and regulatory information	Large models often lead to a combinatorial explosion in parameter estimation	Farquhar et al., 1980; Porcar-Castell et al., 2006; Retkute et al., 2015
Steady-state	Capture the steady-state behavior when internal metabolite concentrations can be assumed to stay constant	Computationally inexpensive; can be used to capture metabolic acclimation	Time-steps typically occur over hours or days; regulatory mechanisms are largely ignored	Cheung et al., 2014; Shaw and Cheung, 2018
Stochastic	Account for a certain unpredictability in the model outcome by considering a probability of occurrence	Can account for randomness, heterogeneity and intrinsic noise	Computationally expensive to run; no single solution	Guerriero et al., 2014; Retkute et al., 2018

*The advantages and limitations of each are discussed and examples of where each type of model has been applied are provided*.

### Empirical vs. Mechanistic Modeling Techniques to Study Photosynthesis

Mathematical modeling within biology is ruled by two paradigms: empirical modeling and mechanistic modeling. Empirical modeling, also known as statistical modeling, fits a model to the data without considering the underlying biological processes (Table 1). Through the observation of a repeated pattern, it is assumed that future events of the same type will result in the same pattern. A straightforward example of an empirical model is the non-rectangular hyperbola of net carbon gas exchange fitted to light response curves (Johnson and Murchie, 2011). While the fit of this model has stood the validation test of time, possible underlying biological mechanisms have only recently been discussed (Retkute et al., 2015; Herrmann et al., 2020). Stegemann et al. (1999) constructed an empirical model relating fluctuating diurnal changes in light intensity to net photosynthesis. By fitting their model parameters to data obtained from two different tree species, they successfully estimated carbon uptake without explaining the mechanisms behind their photosynthetic responses to light.

Empirical models are a powerful tool when the underlying processes are not known and form the premise of machine learning algorithms (Kotsiantis et al., 2007; Angelov and Gu, 2019). The reliability of empirical models improves vastly with the amount of input data available (Kotsiantis et al., 2007), a limitation that is becoming less hindering in the current ‘omics era. Nonetheless, extrapolation of empirical models is difficult and, typically, good predictions cannot be made outside of the range of previously measured values. For example, an empirical model of photosynthesis with parameters fitted to a specific light and temperature regime is unlikely to be transferable to another light and temperature regime, and, instead, the model parameters must be estimated anew (Herrmann et al., 2020).

By contrast, mechanistic models, albeit harder to construct, have several advantages over empirical models (Table 1). Mechanistic models break down a system into smaller components, and the processes by which these components interact with one another are then captured by mathematical equations. Mechanistic models require an in-depth understanding of the system components and their interactions in space and time. However, once a mechanistic model is constructed and its parameters are successfully estimated, few input data are required for outcome prediction. Furthermore, if the same mechanisms apply under different conditions, or outside the range of the initial input values, the model can be applied beyond the range of the initial training data (Geritz and Kisdi, 2012; Ratti, 2018).

Kinetic models of metabolic pathways are examples of mechanistic models: a pathway is broken down into its metabolites and the way in which these metabolites interact with one another can, for example, be described by mass action law or Michaelis-Menten kinetics (Schallau and Junker, 2010). Many successful kinetic models of photosynthesis have been built, and their ability to capture fluctuating light conditions is discussed in the next section. Kinetic models, as with all others mechanistic models, are limited by our knowledge of the system under study.

### Dynamic Modeling to Study Time-Dependent Photosynthetic Responses Under Fluctuating Light

Dynamic models generally encompass time-dependent-models that capture changes over time (Table 1). These models employ a set of ordinary differential equations, or partial differential equations, considering one or more independent variables. Dynamic models can be empirical or mechanistic; however, in biochemistry, dynamic models appear most commonly in the form of kinetic models. Kinetic models of biochemical pathways are mechanistic, dynamic models, as they consider changes in metabolite concentrations over time. Due to a combinatorial explosion of the parameter estimation, dynamic models are generally limited to a handful of equations.

Multiple dynamic models of photosynthesis and the Benson-Calvin cycle reactions exist (Farquhar et al., 1980, 2001; Harley and Tenhunen, 1991; Poolman et al., 2000); however, most of them have not been applied to study fluctuating light acclimation. Kirschbaum et al. (1997) and Pearcy et al. (1997) are among the few to have extended the original Farquhar et al. (1980) model to study fluctuating light regimes over a time frame of seconds to hours. Mott and Woodrow (2000), however, addressed the question of nitrogen resource allocation under fluctuating light regimes by using a much simpler model of rubisco and rubisco activase. In addition, Porcar-Castell et al. (2006) incorporated both regulatory and feedback mechanisms in their dynamic model of PSII, and were able to validate experimentally obtained photochemical and non-photochemical quantum yields under fluctuating light. However, none of these models consider a long-term acclimation of plants to changing light regimes, as they have been parametrized for a much shorter timescale.

Retkute et al. (2015) employed a semi-empirical dynamic model to describe carbon uptake over time as a function of light availability and a constant maximum photosynthetic capacity (Pmax). Pmax is calculated to give the maximum possible carbon uptake over the time-weighted average of a light pattern, representing the acclimation state of the plant. How plants shift from one acclimation state to another, and alter their Pmax accordingly, was discussed by Herrmann et al. (2020) using a time- and temperature-dependent model. However, the sensors and signals that trigger a new photosynthetic acclimated state remain elusive.

As discussed in section Signals Transduction Pathways for Long-Term Acclimation to Fluctuating Light, and highlighted in our previous studies (Dyson et al., 2015; Herrmann et al., 2020), carbon fluxes between the cytosol and the chloroplast seem to be important factors in the photosynthetic acclimation responses of plants. In particular, the resulting changes in sugar vs. starch production have been shown to be crucial for acclimation to different light regimes (Dyson et al., 2015). Modeling the changes in carbon metabolism under fluctuating light could be useful to identify key signals leading to acclimation responses in plants. For instance, using a simple kinetic model and a sensitivity analysis of the model parameters, Nägele and Weckwerth (2014) analyzed the control of sugar homeostasis in plants, and suggested that allosteric effectors alone can account for a considerable readjustment of metabolic homeostasis.

Metabolic control analysis (MCA) quantifies the extent to which fluxes, or concentrations, depend on the model parameters. Thus, when applied to kinetic models, MCA provides a valuable tool for identifying parameters, and thus enzymes, that exert the greatest metabolic control over the fluxes (or species concentrations) in a defined model (ap Rees and Hill, 1994; Poolman et al., 2000). The fact that detailed regulatory information can be included in these dynamic kinetic models represents one of their greater advantages; although at the same time limits the size and complexity over which they can be feasibly solved. Often, a trade-off between the level of mechanistic detail and the feasibility to solve the model is required (Harley and Tenhunen, 1991).

### Steady-State Modeling as a Mechanistic Approach to Study Photosynthetic Acclimation to Fluctuating Light

Photosynthetic acclimation to a sustained change in light regime typically occurs over multiple days. As Athanasiou et al. (2010) observed, a new photosynthetic state is reached only 1 week after a change in exposure from low to high light. Acclimation responses to changes in light regimes are typically not represented by kinetic models, which tend to be parametrized over a timescale of seconds to hours. Instead, genome-scale steady-state models which tend to operate over multiple days and weeks can be used to study photosynthetic acclimation (Table 1; Herrmann et al., 2019a).

Genome-scale, steady-state, metabolic models, employ what are known as constraint-based modeling (CBM) techniques (Lewis et al., 2012), and operate under the assumption that internal metabolite concentrations are constant over time. This approximation is generally valid over longer time-frames, because the changes in metabolic flux leading to new equilibrium states are usually faster when compared to acclimation responses. Whilst these types of models are able to capture the final acclimated steady-state of plant metabolism, they fail to incorporate the mechanisms that initiate and lead to that new steady-state.

More recent variations of CBM techniques aim to overcome these limitations by employing dynamic CBM techniques (Mahadevan et al., 2002; Grafahrend-Belau et al., 2013). Shaw and Cheung (2018), for example, built a dynamic multi-tissue model by using the output of one steady-state model as the input of another steady-state model. By defining a steady-state model at each time point, they were able to effectively analyse resource allocation in plants over days and weeks. The model by Shaw and Cheung (2018) is based on the diel model first published by Cheung et al. (2014), which combined both a day-time (light-dependent) steady-state model and a night-time (light-independent) steady-state model. Dynamic models constructed of steady-state models are thus able to incorporate time-dependent changes, but typically consider changes over a timescale of multiple days.

Different ‘omics datasets can be used to incorporate enzyme regulatory mechanisms into genome-scale stoichiometric models, making it, for instance, possible to study redox changes at a resolution of second to hours with longer process steady-state models (Jamshidi and Palsson, 2010). This kind of analysis, however, has yet to be applied to plants exposed to fluctuating light.

### The Challenges and Opportunities of Using a Holistic Approach to Address Photosynthetic Acclimation to Fluctuating Light

Incorporating data measured over different timescales, and both discrete (e.g., state switching) and continuous scales (e.g., sink metabolite accumulation), poses an immense challenge for the study of photosynthetic acclimation under fluctuating light conditions. In order to incorporate models describing different processes over different periods of times, dimensionality reduction techniques will be necessary. These techniques are designed to reduce model complexity by discarding components that have little effect on the overall outcome of interest (Hummer and Szabo, 2015; Snowden et al., 2017). Successful dimensionality reduction should lead to the identification of essential model components required for predictive power; each fine-tuned according to the timescale over which it must operate, and the magnitude for which can shows significant effects on the system itself.

Purvis et al. (2009) use dimensionality reduction to combine multiple small-scale kinetic models in the human platelet P2Y1 signaling system, and convert it into a single holistic model. Employing known dimensionality reduction techniques (Hummer and Szabo, 2015; Ali Eshtewy and Scholz, 2020) on fast existing kinetic models of photosynthesis, may pave the way for their incorporation into slower process models (such as the genetic changes involved in light acclimation). Unfortunately, neither dimensionality reduction nor model validation techniques are frequently employed in plant sciences. Model validation, both at the experimental and theoretical level (Hasdemir et al., 2014), will need to be done before a model is deemed suitable to be integrated holistically.

Hybrid models, which incorporate both discrete and continuous information, are starting to gain attention in other disciplines (Henzinger, 2000; Bortolussi and Policriti, 2008). A simple analogy for such a hybrid automaton is a thermostat, whereby the law of thermodynamics are described by ordinary differential equations (continuous) but the state of the heater is either on or off (discrete). Thus, we can imagine multiple metabolite concentrations changing on a continuous scale in response to environmental fluctuations, which could emerge in an on/off output response (such as the ones described in sections Signals Transduction Pathways for Long-Term Acclimation to Fluctuating Light and System Outputs: Long-Term Acclimation to Fluctuating Light of the present review, respectively). However, such a system has yet to be identified in plant acclimation to fluctuating light.

The alternative to the mechanistic approaches described above, would be to take an empirical modeling approach. With an ever-increasing and overwhelming amount of multi-omic data available, there are numerous supervised learning algorithms that could be applied to identify “biomarkers” of a given acclimation stage (Mjolsness and DeCoste, 2001; Saeys et al., 2007). While the identification of molecular predictors is promising, these approaches typically do not reveal any information about the mechanisms by which the identified molecules trigger the final acclimated state of the plant. This empirical approach, however, does hold the potential for validating existing hypotheses or generating new hypotheses for experimental validation. For example, if both STN7 and TSP9 were identified as predictors for light acclimation, this would support the idea that TSP9 acts downstream of STN7 in promoting a long-term acclimation response.

Finally, stochastic models can account for random variations in inputs and result in a probability distribution of potential outcomes (Table 1; Guerriero et al., 2014; Retkute et al., 2018). The application of stochastic models to photosynthesis remains limited as of today but holds a great potential for identifying potential thresholds for acclimation. For instance, one could imagine a metabolite concentration that fluctuates in response to environmental changes triggering an acclimation process only once a given threshold concentration is passed. This could be the case of the proposed ROS and carbon-related metabolites discussed above (see section Signals Transduction Pathways for Long-Term Acclimation to Fluctuating Light).

To facilitate the integration of different modeling techniques, rigorously standardized tools are required. For instance, the open platform www.e-photosynthesis.org hosts a collection of dynamic plant models, translated to the Systems Biology Mark-up Language (SBML), and provides a good starting point for any modeler interested in photosynthetic acclimation. Currently, the project consists largely of model parametrized over short timescale, but hopefully it will be extended to longer-time physiological processes in the future, such that an effective integration of the two will be possible.

## Concluding Remarks

As this review has shown, there is a gap between studies that consider short-term responses to changes in light conditions, and those that consider long-term acclimation processes. This gap is evident from both an experimental and a theoretical viewpoint, and is likely the result of the difficulties associated with studying interconnected processes that occur over different timescales. The photosynthetic apparatus is highly complex; thus, understanding the regulatory networks of fluctuating light responses over time will require a deeper understanding of the system itself. This situation is even more complex if we consider that many experimental studies focus on well-defined, non-random changes in irradiance that do not necessarily reflect realistic field conditions (Annunziata et al., 2017).

It is also worth mentioning that, in the present review, we mainly describe how changes in light regimes affects processes at a single-cell level. Under field conditions, as captured in canopy level models, fluctuating light often result in heterogeneous light absorbance across leaves and cells. How these differences at the cellular level are integrated within and across tissues remains an important topic for further consideration in the future, given that this heterogeneity may result in emergent properties that cannot be captured by single-cell models. Emergent properties are those which arise from an interaction of model components, and which cannot be described by either of the components on their own (Bhalla and Iyengar, 1999; Peak et al., 2004; Aderem, 2005). Emergent properties are the reason why it is often difficult to explain the mechanistic basis of empirical models, and are why both approaches are needed to enhance our understanding of photosynthetic acclimation to fluctuating light conditions.

It is well-established that realistic models of complex biological signals will require regulation, feedback signals and non-linear dynamic components (Csete and Doyle, 2002). Identifying potential emergent properties of such systems will require the integration and careful dimensionality reduction of multiple processes (Rascher and Nedbal, 2006). Models will need to be specific enough to capture the individual processes that together lead to emergent system properties but, at the same time, need to be general enough to differentiate noise from signal (Gillespie, 2000; Mélykúti et al., 2010). As a conclusion, a combination of existing and emerging modeling techniques will be required to capture the emergent properties and signaling pathways related to photosynthetic acclimation to fluctuating light in plants.

## Author Contributions

PC, AG, and GJ contributed mainly to the sections System Inputs: Short-Term Responses to Fluctuating Light, System Outputs: Long-Term Acclimation to Fluctuating Light, and Signals Transduction Pathways for Long-Term Acclimation to Fluctuating Light of the present review. HH and J-MS contributed mainly to section Metabolic Modeling in Unraveling the Photosynthetic Acclimation to Fluctuating Light in Plants. All authors co-wrote and approved the manuscript.

## Conflict of Interest

The authors declare that the research was conducted in the absence of any commercial or financial relationships that could be construed as a potential conflict of interest.
